# Early Immunological and Inflammation Proteomic Changes in Elderly COVID-19 Patients Predict Severe Disease Progression

**DOI:** 10.3390/biomedicines13051162

**Published:** 2025-05-10

**Authors:** Shiyang Liu, Wen Xu, Bo Tu, Zhiqing Xiao, Xue Li, Lei Huang, Xin Yuan, Juanjuan Zhou, Xinxin Yang, Junlian Yang, De Chang, Weiwei Chen, Fu-Sheng Wang

**Affiliations:** 1 Medical School of Chinese PLA, Beijing 100853, China; 18811509502@163.com; 2 Senior Department of Infectious Diseases, The Fifth Medical Center of Chinese PLA General Hospital, National Clinical Research Center for Infectious Diseases, Beijing 100073, China; xuwen302yy@163.com (W.X.); tub_302@163.com (B.T.); lixue7796@163.com (X.L.); huangleiwa@sina.com (L.H.); xinyuannatile@sina.com (X.Y.); zhoujuanjuan_302@163.com (J.Z.); yxinxin88@163.com (X.Y.); tianshi427@foxmail.com (J.Y.); 3 Department of Pulmonary and Critical Care Medicine at The Seventh Medical Center, College of Pulmonary and Critical Care Medicine of The Eighth Medical Center, Chinese PLA General Hospital, Beijing 100007, China; Xiao_zq2025@163.com (Z.X.); changde@301hospital.com.cn (D.C.); 4 Yu-Yue Pathology Scientific Research Center, Chongqing 401329, China

**Keywords:** elderly COVID-19 patients, disease progression, biomarkers, inflammation proteomics

## Abstract

**Background:** Elderly patients infected with SARS-CoV-2 are at higher risk of developing cytokine storm and severe outcomes; however, specific immunological and proteomic biomarkers for early prediction remain unclear in this vulnerable group. **Methods:** We enrolled 182 elderly COVID-19 patients from the Chinese PLA General Hospital between November 2022 and April 2023, categorizing them based on progression to respiratory failure requiring mechanical ventilation (defined as severe progression). Olink proteomic analysis was performed on admission serum from 40 propensity score-matched samples, with differentially expressed proteins (DEPs) validated by cytometric bead array (CBA) in 178 patients. To predict severe progression, a model was developed using a 70% training set and validated on a 30% validation set. LASSO regression screened features followed by logistic regression and receiver operating characteristic (ROC) analysis to optimize the model by incrementally incorporating features ranked by random forest importance. **Results:** Elderly patients progressing to severe COVID-19 exhibited early immune dysregulation, including neutrophilia, lymphopenia, monocytopenia, elevated procalcitonin (PCT), C-reactive protein (CRP), interleukin-6 (IL-6), neutrophil-to-lymphocyte ratio (NLR), and systemic immune-inflammation index (SII), as well as coagulation dysfunction and multi-organ injury. Proteomics identified a set of biomarkers, including tumor necrosis factor-related apoptosis-inducing ligand (TRAIL), and revealed disruptions in signaling pathways, including the mTOR and VEGF signaling pathways. The optimal predictive model, which incorporated PCT, IL-6, monocyte percentage, lymphocyte count, and TRAIL, achieved an area under curve (AUC) of 0.870 (0.729–1.000) during validation. TRAIL levels negatively correlated with fibrinogen (*p* < 0.05). **Conclusions:** Elderly COVID-19 patients with severe progression demonstrate early immune dysregulation, hyperinflammation, coagulation dysfunction, and multi-organ injury. The model we proposed effectively predicts disease progression in elderly COVID-19 patients, providing potential biomarkers for early clinical risk stratification in this vulnerable population.

## 1. Introduction

Elderly individuals, characterized by advanced age and comorbidities, represent a high-risk population for SARS-CoV-2 infection and disease progression [1]. According to the US Centers for Disease Control and Prevention (CDC), individuals aged 65–74 face a 60-fold higher risk of severe COVID-19 than those aged 18–29, with the risk escalating to 340-fold for adults over 85. This risk is further amplified in elderly patients with underlying health conditions [2]. Compared to younger adults and children, elderly COVID-19 patients exhibit significantly higher rates of severe disease, intensive care unit (ICU) admission, and mortality [3,4,5]. Therefore, identifying biomarkers for disease severity in elderly patients is crucial for risk prediction and therapeutic target discovery. Although previous studies have proposed biomarkers for COVID-19 severity, research specifically targeting elderly populations remains limited.

Cytokine storm has been identified as a primary driver of severe COVID-19 progression and mortality [6], characterized by significant elevations in IL-2/6/7/10, CXCL10, MCP-1, TNF-α, MIP1α, and G-CSF [7,8,9]. Elderly patients are particularly susceptible to cytokine storm due to age-related immune dysregulation, including impaired T- and B-cell function and excessive inflammatory cytokine production. This may lead to inadequate viral control, prolonged inflammation, and poor outcomes [10]. Proteomic analysis of inflammatory proteins offers a comprehensive approach to understanding cytokine storm mechanisms and identifying predictive biomarkers for disease progression.

Autopsy findings from COVID-19 fatalities have revealed diffuse alveolar damage, a hallmark of acute respiratory distress syndrome (ARDS) [11], for which mechanical ventilation is a critical intervention. A meta-analysis stratified by age demonstrated that ICU mortality rates for mechanically ventilated patients were 47.9% (95% CI: 46.4–49.4%) for those under 40 years, compared to 84.4% (95% CI: 83.3–85.4%) for patients over 80 years [12], highlighting the inferior prognosis of elderly patients progressing to respiratory failure requiring mechanical ventilation. Therefore, we adopted respiratory failure requiring mechanical ventilation as a marker for disease progression to severe status in elderly patients.

This study established a prospective cohort enrolling elderly COVID-19 patients (≥60 years). Based on whether they developed to respiratory failure requiring mechanical ventilation during hospitalization, patients were categorized into severe progression (NS-S) and non-severe progression (NS-N) groups, respectively. Initially, we performed an Olink proteomic analysis focused on inflammatory cytokines in serum at admission on a matched subcohort to identify differentially expressed proteins (DEPs) associated with early disease progression. Subsequently, a predictive model was developed and validated using these DEPs and inflammatory immune markers in a larger cohort.

## 2. Materials and Methods

### 2.1. Study Design and Sample Collection

This prospective study enrolled 182 COVID-19 patients at the Fifth Medical Center and the Seventh Medical Center of the Chinese PLA General Hospital from November 2022 to April 2023. The diagnosis of COVID-19 was determined based on the results of reverse-transcriptase polymerase chain reaction (RT-PCR) or antigen tests of nasopharyngeal swabs, in combination with epidemiological investigations and clinical manifestations. The exclusion criteria were as follows: (1) the presence of tumors or immune system diseases in the progressive stage; (2) immunosuppressant use; (3) a history of major organ transplantation; (4) disease progression due to causes other than COVID-19; (5) progression to respiratory failure requiring mechanical ventilation within 24 h of admission. Patients were categorized into a severe progression group (NS-S, n = 38) and a non-severe progression group (NS-N, n = 144) based on whether they developed respiratory failure requiring mechanical ventilation during their hospital stay. Respiratory failure requiring mechanical ventilation was strictly defined by meeting both criteria: (1) arterial partial pressure of oxygen (PaO_2_) < 60 mmHg on room air, and (2) formal physician order for mechanical ventilation based on clinical assessment. Serum samples were collected from patients within 24 h of admission and stored at −80 °C.

**Figure 1:**
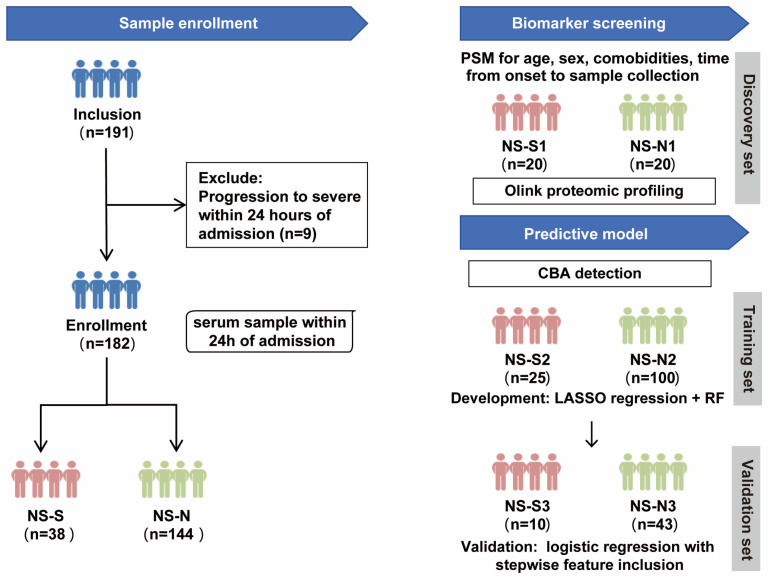
Study design flow chart. Note: The predictive model was developed using 178 patients (from the original cohort of 182), as 4 samples lacked sufficient serum volume for CBA detection. PSM: propensity score matching; CBA: cytometric bead array; RF: random forest.

The study design included two parts (Figure 1). Firstly, to control the influence of confounding factors, we performed propensity score matching (PSM) on a 1:1 basis for age, sex, comorbidities, and time from symptom onset to sample collection between NS-N and NS-S groups of patients to establish a biomarker screening cohort. This resulted in a small sample discovery set of 20 patients per group (NS-N1 and NS-S1) who underwent proteomic profiling to screen for DEPs between the groups. Subsequently, protein quantification of DEPs was conducted on a predictive model cohort including 178 patients (4 excluded because they lacked sufficient serum volume for CBA detection). The cohort was randomly divided into a 70% training set (NS-N2: 100 cases; NS-S2: 25 cases) for model development and a 30% validation set (NS-N3: 43 cases; NS-S3: 10 cases) for predictive performance validation.

We collected personal, clinical, and laboratory information at baseline from the enrolled patients. The personal and clinical information encompassed sex, age, comorbidities, time from onset to sample collection, onset symptom, and vaccination. Immune-related parameters comprised white blood cell (WBC) and main immune cell counts and proportions (lymphocytes, neutrophils, monocytes), procalcitonin (PCT), C-reactive protein (CRP), interleukin-6 (IL-6), neutrophil-to-lymphocyte ratio (NLR), platelet-to-lymphocyte ratio (PLR), lymphocyte-to-monocyte ratio (LMR), and systemic immune-inflammation index (SII, SII = platelet count × neutrophil count/lymphocyte count) [13,14,15]. Other laboratory parameters included coagulation function (platelet count, fibrinogen, D-dimer) and cardiac function (myoglobin, pro-brain natriuretic peptide (BNP), creatine kinase (CK), and lactate dehydrogenase (LDH), liver function (alanine aminotransferase (ALT), aspartate aminotransferase (AST), total bilirubin (TBil)), and renal function (urea, creatinine, uric acid)).

This study was approved by the Ethics Committee of Chinese PLA Hospital (KY-2022-6-44-1), and informed consent was obtained from the patients or their representatives.

### 2.2. Proteomic Profiling of Soluble Factors in Serum

The Olink multiplex proximity extension assay (PEA) inflammation panel, which encompasses 92 inflammation-related proteins (Olink Bioscience AB, Uppsala, Sweden), was employed to quantify serum samples. This innovative PEA technique utilizes pairs of antibodies tagged with oligonucleotides that specifically bind to their corresponding target proteins. A unique polymerase chain reaction (PCR) target sequence is generated via a proximity-dependent DNA polymerization process when these antibodies come into close proximity. This newly formed sequence is then identified and quantified using conventional real-time PCR methods. Data derived from the Olink assay were expressed as normalized protein expression (NPX) levels, paralleling the distributions observed with log2-transformed protein concentrations. After quality control, 76 inflammation-related protein concentrations from 40 patients (biomarker screening cohort) were obtained and analyzed.

### 2.3. Cytometric Bead Array (CBA) Immunoassay

Serum cytokine levels of FMS-like tyrosine kinase 3 ligand (Flt3L), tumor necrosis factor-related apoptosis-inducing ligand (TRAIL), C-X-C motif chemokine ligand 5 (CXCL5), interleukin 12 (IL-12B), monocyte chemoattractant protein 3 (MCP-3), interleukin 24 (IL-24), and interleukin 8 (IL-8) from 178 patients (predictive model cohort) were assessed using QBPlex^®^Multiple Immunoassays for Flow. The serum and a mixture of fluorescent polystyrene microspheres coated with antigen-specific antibodies were incubated to allow cytokines in the serum to bind to the antibodies on the microspheres. Subsequently, a mixture of biotin-labeled antibodies was added to enable their specific binding to the cytokines. Then, Streptavidin-PE was added to facilitate its binding to the biotin. Fluorescence was detected using a flow cytometer, and serum concentrations of cytokines were qualified by referencing the standard curve derived from antigen standards.

### 2.4. Statistical Analysis

Continuous data that conformed to a normal distribution were described as the mean and standard deviation (s.d.) and compared using Student’s *t*-test. Continuous data that did not conform to a normal distribution were described as the median and interquartile range (IQR) and compared using the non-parametric Mann–Whitney U test. Count data were analyzed by using the Chi-square test. To minimize potential bias caused by missing data, multiple imputation using a Markov chain Monte Carlo (MCMC)-based linear regression model was performed to address missing continuous laboratory indicators in the dataset of 178 patients for the development of the predictive model. LASSO regression was performed on the important individual, clinical, and significantly different immune-related indicators, as well as the DEPs. The reduced set of important features determined by lambda min was then ranked using a random forest model. The features ranked by Mean Decrease Accuracy were incrementally added to logistic regression models, employing stepwise receiver operating characteristic (ROC) curve analysis. The data that conformed to a normal distribution were analyzed using Pearson correlation and linear regression, whereas the data that did not conform to a normal distribution were analyzed using Spearman correlation and smoothing spline fits. A two-sided *p* value of <0.05 was considered statistically significant. Bioinformatics analysis of the proteomics data was conducted using R software (version 4.2.1). General statistical analyses were performed using PRISM (version 8) and SPSS (version 27).

## 3. Results

### 3.1. Baseline Information of Elderly COVID-19 Patients

Among the 182 COVID-19 patients, the NS-N group comprised 87 male and 57 female patients, while the NS-S group included 28 male and 10 female patients. The median age of the NS-S group (84 years) was significantly higher than that of the NS-N group (79 years) (*p* = 0.039). The two groups were similar in terms of demographic and clinical characteristics, including sex, comorbidities, time from symptom onset to hospital admission, onset symptoms, and vaccination status. However, the NS-S group exhibited significant differences in immune, coagulation, cardiac, liver, and renal parameters compared to the NS-N group. In terms of immune and inflammation, the NS-S group showed significantly elevated levels of peripheral WBC, neutrophil count, neutrophil percentage, PCT, CRP, IL-6, NLR, and SII (*p* < 0.05). Conversely, the NS-S group exhibited significantly lower lymphocyte count, lymphocyte percentage, and monocyte percentage (*p* < 0.05). In addition, compared to the NS-N group, the NS-S group also showed significantly elevated levels of fibrinogen, D-dimer, myoglobin, BNP, CK, LDH, AST, urea, and creatinine (*p* < 0.05), indicating the disruption of coagulation and injury of cardiac, renal, and liver functions. Comprehensive information can be found in Table 1.

### 3.2. The Serum Proteomic Profile of the Severity Progression of COVID-19 in Elderly Patients

**Figure 2:**
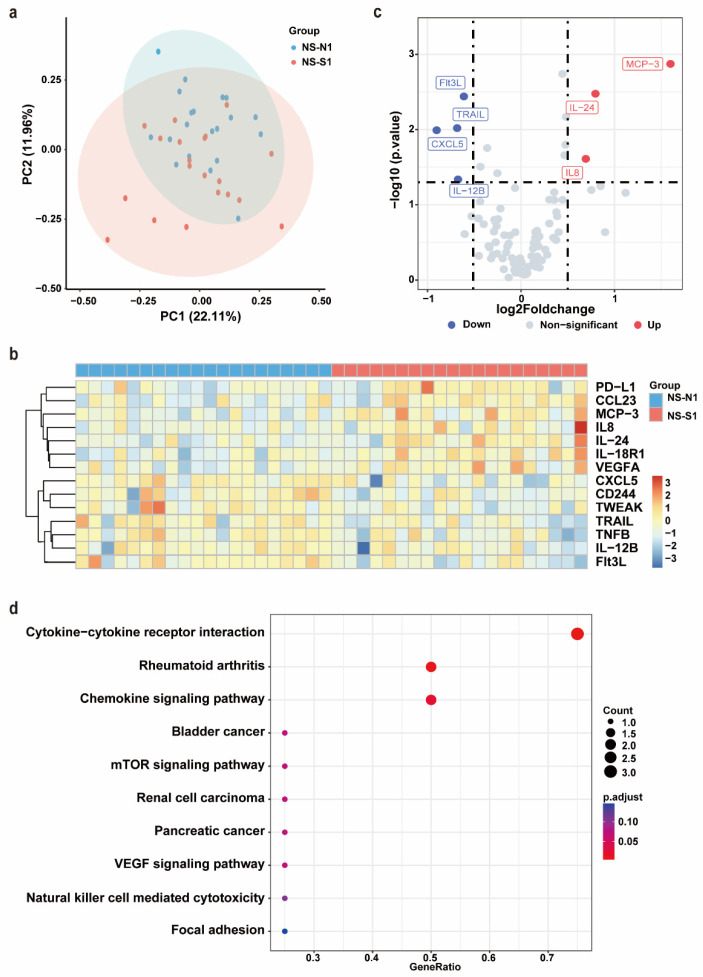
Olink proteomic profiling of NS-N1 and NS-S1 groups. (**a**) PCA of proteins in serum of NS-N1 (blue) and NS-S1 (red) groups at admission. (**b**) The heat map of all DEPs in the NS-N1 and NS-S1 groups. Red: high expression; blue: low expression. Screening criteria for DEPs: *p* < 0.05. (**c**) The volcano plot presents the upregulated (red) and downregulated (blue) DEPs in the NS-N1 and NS-S1 groups. Screening criteria for DEPs: *p* < 0.05, |log2FC| > 0.5. (**d**) Bubble chart of enriched pathways in proteomics by KEGG analysis of DEPs between NS-N1 and NS-S1 groups. Bubble size: protein count; color: adjusted *p* value.

After PSM, no significant differences were observed between the NS-N1 and NS-S1 groups in terms of age, sex, comorbidities, or the time from symptom onset to sample collection (Supplementary Table S1). Serum samples from these 40 patients were subjected to an Olink inflammation panel analysis. Principal component analysis (PCA) and a clustering heat map revealed a discernible separation between the NS-N1 and NS-S1 groups (Figure 2a,b). Compared to the NS-N1 group, the NS-S1 group exhibited significant upregulation of seven inflammatory proteins and downregulation of seven inflammatory proteins. Using a threshold of *p* < 0.05 and |log2FC| > 0.5, seven DEPs were identified. Among these, Flt3L, TRAIL, CXCL5, and IL-12B were significantly downregulated in the NS-S1 group, while MCP-3, IL-24, and IL-8 were significantly upregulated (Figure 2c). KEGG pathway enrichment analysis indicated that the DEPs were primarily enriched in pathways related to inflammatory response regulation (e.g., cytokine–cytokine receptor interaction, chemokine signaling pathway) and immune modulation (e.g., mTOR signaling pathway, VEGF signaling pathway) (Figure 2d).

### 3.3. The Development and Validation of a Predictive Model for the Severity Progression of COVID-19 in Elderly Patients

**Figure 3:**
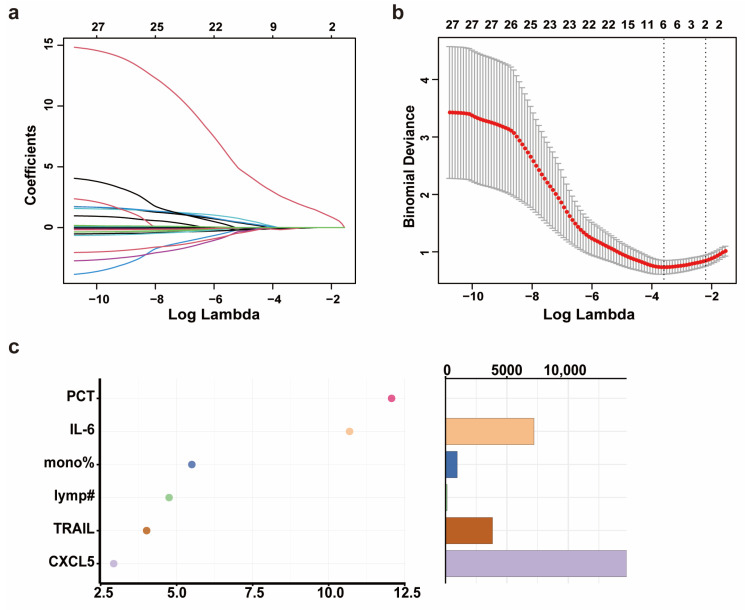
The development and validation of a predictive model for the severity progression of COVID-19 in elderly patients. (**a**) The coefficient path plot of LASSO regression. Each colored curve represents the trajectory of a corresponding feature’s coefficient across the regularization path, with distinct line colors differentiating individual variables. The x-axis shows the log-transformed lambda values (log(lambda)). (**b**) The cross-validation error plot of LASSO regression. The x-axis shows the log-transformed lambda values (log(lambda)). The y-axis shows the binomial deviance (mean ± standard error). The two vertical dotted lines represent lambda min (left, the value of lambda that gives the minimum mean cross-validated error) and lambda 1SE (right, the largest value of lambda such that the error is within 1 standard error of the minimum). (**c**) The importance ranking (left) and relative abundance (right) of filtered features based on Mean Decrease Accuracy calculated by random forest. Higher Mean Decrease Accuracy values (x-axis in left plot) indicate stronger predictive importance. The random forest model was implemented with 1000 trees (number of trees = 1000) and 3 variables tried at each split. Note: mono% means monocyte percentage; lymp# means lymphocyte count.

Through the analysis of the propensity score-matched biomarker screening cohort, we identified Flt3L, TRAIL, CXCL5, IL-12B, MCP-3, IL-24, and IL-8 as candidate biomarkers for severe progression of COVID-19 in elderly patients. Subsequently, we quantified the levels of the seven DEPs in the serum of 178 patients (predictive model cohort) using CBA (Supplementary Table S2). Prior to predictive model development, we addressed missing data for PCT and CRP through MCMC multiple imputation. In the training set (NS-N2: 100 cases; NS-S2: 25 cases), we included variables such as age, sex, comorbidities, days from symptom onset to sample collection, WBC, neutrophil percentage, neutrophil count, lymphocyte percentage, lymphocyte count, monocyte percentage, PCT, CRP, IL-6, Flt3L, IL-8, IL-12B, TRAIL, CXCL5, IL-24, and MCP3. LASSO regression was used to filter features. The optimal regularization parameter λ for LASSO regression was 0.027, which retained six features according to lambda min while compressing the coefficients of the remaining features to zero (Figure 3a,b). To address potential overfitting (limited remaining features and small sample size of NS-S2 and NS-S3), we employed a two-step approach: (1) random forest-based feature importance ranking, followed by (2) logistic regression with incremental feature addition (for β coefficient of each biomarker, see Supplementary Table S3). In the validation set, model robustness was assessed by stepwise ROC curve analysis.

**Figure 4:**
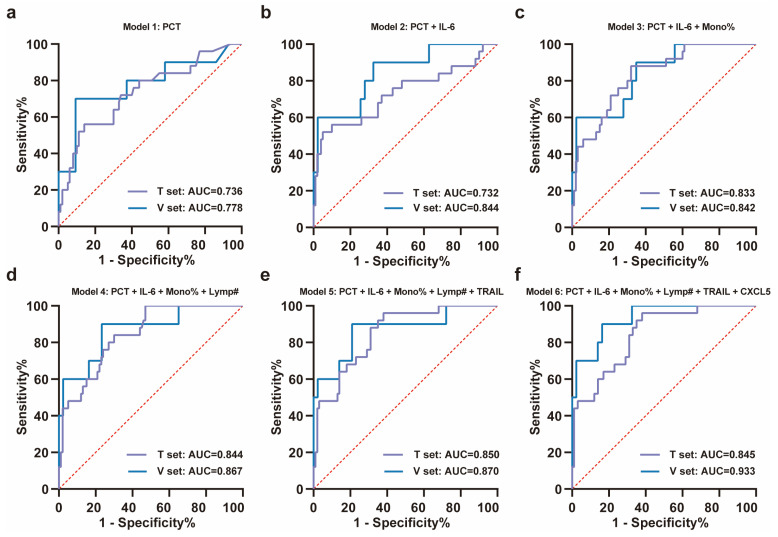
ROC analysis of training set and validation set for the severity progression of COVID-19. (**a**–**f**) The stepwise ROC curves plotted in the training and validation sets according to the importance ranking of features. The ROC curves for (**a**) PCT; (**b**) PCT + IL-6; (**c**) PCT + IL-6 + monocyte percentage; (**d**) PCT + IL-6 + monocyte percentage + lymphocyte count; (**e**) PCT + IL-6 + monocyte percentage + lymphocyte count + TRAIL; (**f**) PCT + IL-6 + monocyte percentage + lymphocyte count + TRAIL + CXCL5. All ROC curves were plotted with the red dashed diagonal line (AUC = 0.5) as reference. Note: T set means training set, V set means validation set, mono% means monocyte percentage, and lymp# means lymphocyte count.

Using a random forest model, the importance of these features was ranked as PCT, IL-6, monocyte percentage, lymphocyte count, TRAIL, and CXCL5 according to Mean Decrease Accuracy (Figure 3c). ROC curves were plotted by sequentially adding each feature to the model (Figure 4a–f). The results demonstrated that the value of AUC reached its maximum after the inclusion of the fifth feature, TRAIL. In the validation set (NS-N3: 43 cases; NS-S3: 10 cases), the model’s sensitivity and accuracy reached their optimal levels, while the AUC and specificity also performed well after TRAIL was included (Figure 4d, Table 2). These findings underscored the critical value of TRAIL in predicting severe progression of COVID-19 in elderly patients. We further validated the stability of the model in stratified analyses by age and sex (Supplementary Table S4).

### 3.4. Correlation Analysis Between the Novel Biomarker, TRAIL, and Clinical Laboratory Parameters

As a member of the Tumor Necrosis Factor Superfamily (TNFSF), TRAIL induces apoptosis through a death domain and is critical for immune regulation. The expression of TRAIL has been reported on the surface of activated NK and T cells, especially in antitumor activity. TRAIL has been shown to induce apoptosis of tumor cells through its interaction with death receptors TRAIL-R1 and TRAIL-R2, but TRAIL-R4 can act as a decoy receptor, inhibiting TRAIL-induced apoptosis and contributing to tumor cell survival [16]. Although TRAIL has been extensively studied in the context of cancer, its low levels have also been suggested to be associated with COVID-19 severity [17].

**Figure 5:**
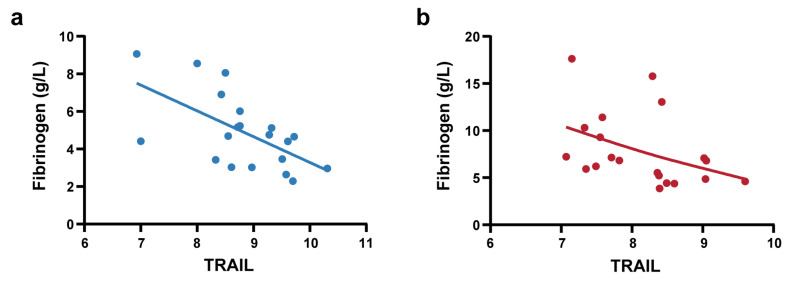
Correlation analysis between TRAIL and fibrinogen. (**a**) Pearson correlation analysis between Olink-detected TRAIL and fibrinogen in NS-N1 group (r = −0.603, *p* = 0.005). The blue line represents linear regression (R square = 0.363). (**b**) Spearman correlation analysis between Olink-detected TRAIL and fibrinogen in the NS-S1 group (ρ = −0.555, *p* = 0.011). The red line represents a smoothing spline fit (knots = 5) to illustrate the trend.

To further elucidate the clinical significance of TRAIL, we conducted correlation analysis between TRAIL levels and clinical laboratory parameters that exhibited significant differences between the NS-N and NS-S groups (Supplementary Table S5). The results demonstrated that in the predictive model cohort, TRAIL showed significant and negative correlations with fibrinogen (Spearman’s ρ = −0.345, *p* < 0.001) and LDH (Spearman’ ρ = −0.181, *p* < 0.05). We also performed correlation analysis in the biomarker screening cohort of 40 patients (PSM) to control the influence of confounding factors. We found that Olink-detected TRAIL exhibited significant strong negative correlations with fibrinogen (Spearman’s ρ = −0.627, *p* < 0.001). Stratified analyses were conducted in the NS-N1 and NS-S1 subgroups. Notably, these correlations of TRAIL and fibrinogen were consistently observed in the NS-N1 and NS-S1 subgroups (Figure 5a,b).

## 4. Discussion

Advanced age is a significant risk factor for severe disease progression and mortality in COVID-19 patients [1,3]. Age-related changes, including altered inflammatory responses, diminished innate immunity, and impaired adaptive immunity [18], may exacerbate inflammatory responses upon SARS-CoV-2 infection, increasing the likelihood of cytokine storm, a key driver of severe disease and death. In this study, we observed early immune dysregulation and hyperinflammation in elderly COVID-19 patients with severe progression, accompanied by coagulation, cardiac, hepatic, and renal dysfunction. Through proteomic analysis, we identified a set of candidate biomarkers (Flt3L, TRAIL, CXCL5, IL-12B, MCP-3, IL-24, and IL-8). The predictive model based on PCT, IL-6, monocyte percentage, lymphocyte count, and TRAIL was developed and validated, which demonstrated strong predictive value. TRAIL, a novel biomarker for disease severity, also showed significant correlations with fibrinogen.

Our results showed that elderly COVID-19 patients exhibited early signs of severe progression, such as immune cell alterations (increased neutrophil count and percentage, decreased lymphocyte percentage, and elevated NLR), consistent with previous studies [19,20,21,22]. On the one hand, SARS-CoV-2 infection activates and recruits neutrophils to infection sites, where they release inflammatory mediators that exacerbate tissue damage [23]. However, during early disease progression, we observed discordant serum levels of two key neutrophil chemokines—IL-8 and CXCL5. Elevated IL-8 levels align with the massive neutrophil recruitment seen in severe progression, whereas reduced CXCL5 may reflect age-related differences in our cohort or early-stage disease dynamics. This divergence underscores the complexity of neutrophil migration regulation. On the other hand, SARS-CoV-2 virus could directly infect lymphocytes or induce lymphocyte apoptosis via inflammatory factors, reducing lymphocyte number and weakening their antiviral responses [20]. Additionally, we also observed that decreased serum IL-12B in the severe progression group. As IL -12B is crucial for Th1 cell differentiation and proliferation [24], its reduction may impair the T-cell killing function. These alterations align with the pathogenesis of cytokine storm in severe COVID-19, characterized by hyperactivated innate immunity and delayed adaptive immunity [6].

Elderly COVID-19 patients showed early monocyte depletion during severe progression. We observed decreased circulating monocyte percentages in early-stage deterioration, accompanied by elevated MCP-3 levels—a chemokine responsible for monocyte recruitment to inflammatory sites. Autopsy findings confirmed substantial monocyte/macrophage infiltration in lung tissues of severe cases [25]. Mechanistically, SARS-CoV-2 E protein induces the NF-κB signaling pathway and promotes MCP-3 gene transcription in airway epithelial cells [26], mediating massive monocyte migration to the lungs, thereby reducing peripheral monocyte pools. Additionally, SARS-CoV-2 directly infects monocytes through Fcγ receptors, triggering pyroptosis and exacerbating systemic inflammation [27].

Impaired innate antiviral immunity may represent a critical mechanism underlying severe progression in elderly COVID-19 patients. We observed decreased serum Flt3L levels in the early stage of severe disease. Flt3L enhances hematopoietic stem cell activation and CD8α+ dendritic cell development, boosting antiviral immunity [28]. Downregulation of Flt3L reflects immune imbalance and suppressed antiviral capacity of innate immunity during the early stage of severe progression in elderly COVID-19 patients. Furthermore, the enrichment of DEPs in the mTOR signaling pathway was identified, consistent with a previous study [29]. In MERS-CoV infection, inhibition of the mTOR pathway prior to infection resulted in a 60% inhibition of virus replication in epithelial-like cells [30]. However, as the modulation of cellular pathways of SARS-CoV-2 is similar to that of MERS-CoV, further research is still needed.

Hyperinflammation of elderly COVID-19 patients with severe progression is closely linked to coagulation and vascular endothelial cell damage. Elevated IL-6, a key cytokine in cytokine storm, promotes inflammation and induces acute-phase proteins (e.g., CRP, fibrinogen, and complement), activates coagulation cascades, and exacerbates disseminated intravascular coagulation [31]. This aligns with our findings of increased IL-6, fibrinogen, and D-dimer levels in severe cases. VEGF signaling pathway enrichment suggests pulmonary endothelial barrier disruption, with elevated VEGF indicating increased vascular permeability and alveolar accumulation of inflammatory factors, an early sign of lung injury [32].

Furthermore, we observed that SARS-CoV-2 infection induces acute multi-organ damage in elderly patients at the early stage of severe progression. Previous studies have also reported a significantly higher incidence of acute multi-organ injury in severe COVID-19 cases. This may be attributed to the widespread distribution of the SARS-CoV-2 receptor, angiotensin-converting enzyme 2 (ACE2), across multiple organs and cells in the human body, enabling the virus to infect the kidneys, heart, liver, and other organs. Histopathological evidence of regional viral replication, supported by the detection of viral RNA, further substantiates the multi-organ involvement in COVID-19 [33,34]. Additionally, the prevalence of pre-existing multi-organ dysfunction in elderly individuals may exacerbate acute organ damage following viral infection [35].

It was reported in this study that the marked downregulation of serum TRAIL in elderly patients with disease progression aligns with a previous study [36]. TRAIL signaling represents a complex biological mechanism with context-dependent effects. On one hand, reduced TRAIL levels may inhibit TRAIL-induced apoptosis of SARS-CoV-2-infected cells, potentially promoting viral persistence and enhanced inflammatory responses [37]. On the other hand, TRAIL’s immunomodulatory effects are mediated through its receptors. The binding of TRAIL to its functional receptors can activate both NF-κB and MAPK signaling pathways [38], suggesting that its downregulation may impair both immune regulation and antiviral responses. Furthermore, the relative expression levels of TRAIL and its decoy receptors (particularly TRAIL-R4) may critically determine its biological effect on SARS-CoV-2-infected cells. This receptor balance (e.g., TRAIL:TRAIL-R4 ratio) warrants investigation in future studies as a potential modulator of COVID-19 severity. Additionally, we identified significant negative correlation between TRAIL and fibrinogen. This finding suggests a potential pathophysiological mechanism wherein impaired TRAIL-mediated apoptosis of virus-infected cells may exacerbate inflammatory responses, subsequently triggering hepatic fibrinogen overproduction—particularly in COVID-19 patients with severe progression. Consistently, higher fibrinogen has been previously associated with COVID-19 severity [39,40]. While this relationship seems contradictory to a previous study suggesting TRAIL to be a target of thrombin [41], our finding nevertheless suggests that TRAIL may function as a novel regulatory node connecting inflammatory and coagulative pathways in SARS-CoV-2 infection. We also observed discrepancies in the correlations between TRAIL and certain clinical parameters (e.g., urea, AST, D-dimer) across the biomarker screening cohort and predictive model cohort (Table S3). These discrepancies may stem from differences in sample sizes, assay sensitivity, and inherent biological variability.

To predict disease progression in elderly COVID-19 patients, we developed a prognostic model through a prospective cohort study to identify early indicators of severe outcomes and provide clues for clinical decision-making. Our model offers three key advantages: (1) integrating immunological and inflammatory proteomic profiling to elucidate mechanisms underlying cytokine storm-driven severe progression and mortality; (2) employing PSM to control for confounders in the biomarker screening cohort, followed by feature selection (LASSO regression) and importance ranking (random forest) to optimize predictor inclusion; and (3) demonstrating robust performance in the validation set, with a high AUC (0.870), sensitivity (0.900), specificity (0.791), and accuracy (0.906). Notably, we identified TRAIL as a novel biomarker with potential utility for risk stratification in this vulnerable population. Additionally, among patients who progressed to severe disease, the median time from admission to mechanical ventilation was 2 [1,2,3,4] days. These findings suggest that admission biomarker levels may help identify high-risk patients early enough for intervention (e.g., closer monitoring or pre-emptive therapy).

There are a few limitations of this study. First, our study focused on elderly patients of Asian ethnicity in China. Therefore, future multi-center and large-scale studies should validate our findings and assess whether our model can be generalized to patients in other age groups and other ethnic groups. Second, pre-admission medication and vaccination data were not systematically collected, and unmeasured confounders (e.g., genetic/environmental factors) limited our assessment of their effects on disease progression and biomarkers. Future studies should include these factors to clarify their roles. Furthermore, our study provided a correlation between TRAIL and fibrinogen; however, further studies are needed to explore whether and how TRAIL participates in SARS-CoV-2 pathogenesis and influences fibrinogen expression. Finally, as our study was conducted during the Omicron BA.5.2/BF.7-dominant period in China, so further comparative studies with other SARS-CoV-2 variants or respiratory viral infections are needed to determine whether the identified proteomic characteristics are strain-specific or represent broader pathogenic mechanisms.

## 5. Conclusions

Elderly patients progressing to severe COVID-19 show early immune dysregulation, hyperinflammation, coagulation issues, and multi-organ injury, which indicate the mechanisms of cytokine storm characterized by hyperactivated innate immunity and delayed adaptive immunity, as well as the virus-induced damage to blood vessels and organs during SARS-CoV-2 infection and replication. The model we proposed, incorporating PCT, IL-6, monocyte percentage, lymphocyte count, and TRAIL, effectively predicts disease progression in elderly COVID-19 patients. Importantly, reduced TRAIL levels may inhibit TRAIL-induced apoptosis and promote the persistence of virus-infected cells, thereby enhancing inflammatory responses, which provides a novel potential target for early prediction and intervention of respiratory failure correction or severe disease progression control in elderly patients with COVID-19.

## Figures and Tables

**Table 1:** Baseline characteristics of study patients.

Characteristics	NS-N (n = 144)	NS-S (n = 38)	*p* Value
**Personal information**			
Sex			
Male	87 (60.42%)	28 (73.68%)	0.131
Female	57 (39.58%)	10 (26.32%)	
Age (years) *	79.00 (69.00, 89.00)	84.00 (75.00, 88.00)	0.039
Comorbidities			
Circulatory system diseases	104 (72.22%)	27 (71.05%)	0.886
Endocrine system diseases	31 (21.53%)	7 (18.42%)	0.675
Digestive system diseases	40 (27.78%)	8 (21.05%)	0.403
Nervous System diseases	27 (18.75%)	9 (23.68%)	0.497
Urinary system diseases	29 (20.14%)	10 (26.32%)	0.409
Respiratory system diseases	19 (13.19%)	3 (7.89%)	0.576
Musculoskeletal system diseases	11 (7.64%)	4 (10.53%)	0.521
Other diseases	19 (13.19%)	3 (7.89%)	0.576
**Clinical information**			
Time from onset to sample collection (days)	7.00 (3.00, 10.00)	6.00 (4.00, 8.00)	0.458
Onset symptoms			
Fever	120 (83.33%)	32 (84.21%)	0.897
Cough	106 (73.61%)	32 (84.21%)	0.175
Expectoration	89 (61.81%)	24 (63.16%)	0.879
Sore throat	46 (31.94%)	11 (28.95%)	0.723
Others	86 (59.72%)	27 (71.05%)	0.200
Vaccination			
Unvaccinated	21 (14.58%)	5 (13.16%)	0.726
Vaccinated	26 (18.06%)	5 (13.16%)	
Unknown	97 (67.36%)	28 (73.68%)	
**Immune-related parameters**			
WBC (10^9^/L) **	4.84 (3.80, 6.73)	6.31 (4.78, 8.12)	0.007
Neutrophils (%) ***	70.10 (58.25, 81.40)	83.65 (76.80, 87.70)	<0.001
Lymphocytes (%) ***	18.70 (10.45, 28.65)	10.50 (6.10, 16.50)	<0.001
Monocytes (%) ***	8.10 (5.25, 11.25)	5.00 (2.70, 7.60)	<0.001
Neutrophils (10^9^/L) ***	3.42 (2.40, 5.09)	5.06 (3.91, 7.11)	<0.001
Lymphocytes (10^9^/L) ***	0.95 (0.65, 1.27)	0.66 (0.43, 0.90)	<0.001
Monocytes (10^9^)/L)	0.40 (0.27, 0.56)	0.29 (0.18, 0.56)	0.082
PCT (ng/mL) ***	0.06 (0.03, 0.10)	0.24 (0.08, 0.64)	<0.001
CRP (mg/L) *	16.09 (4.50, 44.20)	51.15 (5.00, 88.40)	0.023
IL-6 (pg/mL) ***	18.28 (6.33, 43.24)	51.98 (20.45, 188.40)	<0.001
NLR ***	3.65 (1.94, 7.60)	7.78 (4.60, 14.63)	<0.001
PLR	180.11 (123.44, 278.05)	226.67 (137.50, 358.62)	0.350
LMR	2.29 (1.58, 3.73)	2.10 (1.10, 3.50)	0.120
SII **	625.80 (331.02, 1258.00)	1505.06 (597.57, 2024.66)	0.004
**Coagulation-related parameters**			
Platelet (10^9^/L) *	165.00 (133.00, 214.00)	144.00 (121.00, 188.00)	0.010
Fibrinogen (g/L) **	4.65 (3.52, 6.49)	5.80 (4.43, 7.64)	0.002
D-dimer (mg/L) *	0.49 (0.24, 0.96)	0.84 (0.32, 1.75)	0.010
**Cardiac-related parameters**			
Myoglobin (ng/mL) ***	48.37 (31.67, 90.55)	141.60 (68.33, 196.40)	<0.001
BNP (pg/mL) ***	322.90 (108.00, 762.20)	613.70 (387.00, 2953.00)	<0.001
CK (U/L) **	77.00 (50.00, 135.00)	131.00 (68.00, 360.00)	0.002
LDH (U/L) ***	227.00 (192.00, 266.00)	283.00 (251.00, 368.00)	<0.001
**Liver-related parameters**			
ALT (U/L)	20.00 (16.00, 29.00)	21.00 (16.00, 30.00)	0.429
AST (U/L) ***	27.00 (22.00, 37.00)	39.00 (30.00, 49.00)	<0.001
TBil (μmol/L)	9.50 (6.60, 12.90)	11.20 (7.80, 13.60)	0.136
**Renal-related parameters**			
Urea (mmol/L) ***	5.60 (4.50, 7.40)	8.20 (6.24, 10.60)	<0.001
Creatinine (μmol/L) ***	77.00 (61.00, 91.00)	99.00 (73.00, 118.00)	<0.001
Uric acid (μmol/L)	270.40 (215.00, 336.00)	288.00 (204.00, 347.00)	0.773

Note: The number of cases in the NS-N and NS-S groups are as follows: fibrinogen (143 vs. 38), D-dimer (140 vs. 37), myoglobin/PCT/CK (139 vs. 37), BNP (137 vs. 38), CRP (134 vs. 36), ALT (129 vs. 35), AST/TBil/urea/creatinine/uric acid (143 vs. 37), and LDH (142 vs. 37). No missing values were observed for other characteristics. * means *p* < 0.05; ** means *p* < 0.01; *** means *p* < 0.001. Abbreviations: WBC, white blood cell; PCT, procalcitonin; CRP, C-reactive protein; IL-6, interleukin-6; NLR, neutrophil-to-lymphocyte ratio; PLR, platelet-to-lymphocyte ratio; LMR, lymphocyte-to-monocyte ratio; SII, systemic immune-inflammation index; BNP, pro-brain natriuretic peptide; CK, creatine kinase; LDH, lactate dehydrogenase; ALT, alanine aminotransferase; AST, aspartate aminotransferase; TBil, total bilirubin.

**Table 2:** Performance of predictive models for severe disease progression of elderly COVID-19 patients in training and validation sets.

Model	Features	Set	AUC (95%CI)	Sensitivity	Specificity	Accuracy
1	PCT	Training	0.736 (0.620, 0.851)	0.560	0.860	0.808
		Validation	0.778 (0.591, 0.965)	0.700	0.907	0.849
2	PCT + IL-6	Training	0.732 (0.602, 0.862)	0.520	0.950	0.848
		Validation	0.844 (0.707, 0.981)	0.600	0.977	0.887
3	PCT + IL-6 + mono%	Training	0.833 (0.749, 0.916)	0.880	0.680	0.856
		Validation	0.842 (0.708, 0.975)	0.600	0.977	0.867
4	PCT + IL-6 + mono% + lymp#	Training	0.844 (0.767, 0.921)	0.840	0.700	0.848
		Validation	0.867 (0.736, 0.999)	0.900	0.767	0.887
5	PCT + IL-6 + mono% + lymp# + TRAIL	Training	0.850 (0.772, 0.927)	0.960	0.620	0.864
		Validation	0.870 (0.729, 1.000)	0.900	0.791	0.906
6	PCT + IL-6 + mono% + lymp# + TRAIL + CXCL5	Training	0.845 (0.766, 0.924)	0.960	0.620	0.872
		Validation	0.933 (0.857, 1.000)	0.900	0.873	0.906

Note: mono% means monocyte percentage; lymp# means lymphocyte count.

## Data Availability

The data used to support the findings of this study are available from the corresponding author upon reasonable request.

## Supplementary Materials

The following supporting information can be downloaded at https://www.mdpi.com/article/10.3390/biomedicines13051162/s1, Table S1: Baseline characteristics of NS-N1 and NS-S1 groups; Table S2: Cytokine levels of NS-N and NS-S groups detected by CBA; Table S3: Logistic regression β coefficients of predictive models using the training set for the severe disease progression of elderly COVID-19 patients with incremental feature inclusion; Table S4: Performance of predictive models stratified by age and sex for the severe disease progression of elderly COVID-19 patients; Table S5: Correlations between TRAIL and laboratory parameters.
