# Supplementary material for: Early Immunological and Inflammation Proteomic Changes in Elderly COVID-19 Patients Predict Severe Disease Progression
Source: Biomedicines. 2025 May 10;13(5):1162. doi: 10.3390/biomedicines13051162 (PMC12108611; doi:10.3390/biomedicines13051162)
Supplement: Supplementary file 1 [file biomedicines-13-01162-s001.zip › biomedicines-3588289-supplementary.pdf]

Supplementary Materials

Supplementary Table S1. Baseline characteristics of NS-N1 and NS-S1 groups.

Characteristics	NS-N1 (n = 20)	NS-S1 (n = 20)	*p* value
Personal information			
Sex			
Male	15.00(75.00%)	18.00(90.00%)	0.407
Female	5.00(25.00%)	2.00(10.00%)	
Age (years)	85.00(74.00,94.00)	84.00(77.00,95.00)	0.989
Comorbidities			
Circulatory system diseases	15.00(75.00%)	15.00(75.00%)	1.000
Endocrine system diseases	4.00(20.00%)	4.00(20.00%)	1.000
Digestive system diseases	5.00(25.00%)	7.00(35.00%)	0.490
Nervous system diseases	1.00(5.00%)	2.00(10.00%)	0.548
Urinary system diseases	0.00(0.00%)	2.00(10.00%)	0.147
Respiratory system diseases	1.00(5.00%)	3.00(15.00%)	0.292
Musculoskeletal system diseases	4.00(20.00%)	5.00(25.00%)	0.705
Others	0.00(0.00%)	2.00(10.00%)	0.147
Clinical information			
Time from the onset to sample collection (days)	4.00(2.00,10.00)	6.00(4.00,8.00)	0.565
Onset symptoms			
Fever	17.00(85.00%)	16.00(80.00%)	1.000
Cough	5.00(25.00%)	4.00(20.00%)	1.000
Expectoration	12.00(60.00%)	17.00(85.00%)	0.077
Sore throat	8.00(40.00%)	11.00(55.00%)	0.342
Others	11.00(55.00%)	11.00(55.00%)	1.000
Vaccination			
Unvaccinated	4.00(20.00%)	2.00(10.00%)	0.794
Vaccinated	5.00(25.00%)	4.00(20.00%)	
Unclear	11.00(55.00%)	14.00(70.00%)	

Supplementary Table S2. Cytokine levels of NS-N and NS-S groups detected by CBA.

Characteristics	NS-N (n = 143)	NS-S (n = 35)
Flt3L	3.03(2.27,4.05)	2.81(2.38,4.05)
TRAIL	25.37(14.27,45.79)	19.65(14.27,31.39)
CXCL5	92.48(35.31,182.71)	49.81(21.34,97.21)
IL-12B	7.33 ± 4.95	6.80 ± 2.68

MCP-3	13.51 ± 2.70	13.90 ± 2.37
IL-24	8.11(5.76,12.30)	8.92(5.76,11.44)
IL-8	9.83(4.90,21.96)	18.46(9.45,44.08)

Supplementary Table S3. Logistic regression β coefficients of the predictive models using the training set for the severe disease progression of elderly COVID-19 patients with incremental feature inclusion.

Model	PCT	IL-6	Mono%	Lymp#	TRAIL	CXCL5
1	0.499					
2	0.271	0.008				
3	0.202	0.008	-0.188			
4	0.205	0.007	-0.159	-0.433		
5	0.225	0.007	-0.146	-0.510	-0.016	
6	0.231	0.007	-0.153	-0.521	-0.017	<0.001

Note: mono% means monocyte percentage; lymp# means lymphocyte count.

Supplementary Table S4. Performance of predictive models stratified by age and sex for severe disease progression of elderly COVID-19 patients.

Model 5	AUC（95%CI)	Sensitivity	Specificity	Accuracy
Overall	0.850(0.772,0.927)	0.960	0.620	0.864
Age				
≤ 79 years	0.882(0.767,0.997)	0.875	0.852	0.887
> 79 years	0.807(0.684,0.930)	0.696	0.824	0.825
Sex				
Male	0.820(0.712,0.928)	0.941	0.635	0.825
Female	0.929(0.854,1.000)	0.875	0.892	0.844

Note: Models were developed in the training set, and the optimal model (Model 5) was used to compare predictive performance.

Supplementary Table S5. Correlations between TRAIL and laboratory parameters.

Laboratory parameters	CBA-detected TRAIL (n = 178)		Olink-detected TRAIL (n = 40)	
	ρ	*p* value	ρ	*p* value
Platelet count	-0.017	0.818	0.079	0.630
Fibrinogen[ab]	-0.345	<0.001	-0.627	<0.001
D-dimer	0.106	0.159	-0.166	0.306
Myoglobin	-0.028	0.714	-0.116	0.476
BNP	-0.041	0.587	-0.238	0.140
CK	0.034	0.651	0.021	0.896
LDH[ab]	-0.181	0.015	-0.370	0.019
AST	0.133	0.076	0.302	0.058
Urea[b]	-0.117	0.120	-0.403	0.010
Creatinine	0.056	0.459	-0.274	0.087

Note: a, significant correlation between CBA-detected TRAIL and laboratory parameters; b: significant correlation between Olink-detected TRAIL and laboratory parameters.
